# Silver Nanoparticles Loaded on Polyethylene Terephthalate Films Grafted with Chitosan

**DOI:** 10.3390/polym15010125

**Published:** 2022-12-28

**Authors:** Guadalupe Gabriel Flores-Rojas, Felipe López-Saucedo, Ricardo Vera-Graziano, Héctor Magaña, Eduardo Mendizábal, Emilio Bucio

**Affiliations:** 1 Departamento de Química e Ingeniería Química, Centro Universitario de Ciencias Exactas e Ingenierías, Universidad de Guadalajara, Blvd. M. García Barragán # 1451, Guadalajara 44430, Jalisco, Mexico; 2 Departamento de Química de Radiaciones y Radioquímica, Instituto de Ciencias Nucleares, Universidad Nacional Autónoma de México, Circuito Exterior, Ciudad Universitaria, Mexico City 04510, Mexico; 3 Instituto de Investigaciones en Materiales, Universidad Nacional Autónoma de México, Circuito Exterior, Ciudad Universitaria, Mexico City 04510, Mexico; 4 Facultad de Ciencias Químicas e Ingeniería, Universidad Autónoma de Baja California, Calzada Universidad # 14418, Parque Industrial Internacional Tijuana, Tijuana 22390, Mexico

**Keywords:** chitosan, silver, nanoparticles, polyethylene terephthalate, graft, coating

## Abstract

Currently, polyethylene terephthalate (PET) is one of the most widely used polymeric materials in different sectors such as medicine, engineering, and food, among others, due to its benefits, including biocompatibility, mechanical resistance, and tolerance to chemicals and/or abrasion. However, despite all these excellent characteristics, it is not capable of preventing the proliferation of microorganisms on its surface. Therefore, providing this property to PET remains a difficult challenge. Fortunately, different strategies can be applied to remove microorganisms from the PET surface. In this work, the surface of the PET film was functionalized with amino groups and later with a dicarboxylic acid, allowing a grafting reaction with chitosan chains. Finally, the chitosan coating was loaded with silver nanoparticles with an average size of 130 ± 37 nm, presenting these materials with an average cell viability of 80%. The characterization of these new PET-based materials showed considerable changes in surface morphology as well as increased surface hydrophilicity without significantly affecting their mechanical properties. In general, the implemented method can open an alternative pathway to design new PET-based materials due to its good cell viability with possible bacteriostatic activity due to the biocidal properties of silver nanoparticles and chitosan.

## 1. Introduction

The presence and proliferation of microorganisms as biofilm [1] on polymeric materials for medical use and food containers can cause adverse effects on healthcare as well as for the stored food [2,3]. Therefore, polymeric materials capable of inhibiting the proliferation of microorganisms have gained great relevance. Therefore, it is necessary to develop more durable and efficient materials capable of inhibiting the proliferation not only of bacteria but also of fungi and even viruses [4] that can cause infections in open wounds, as well as avoiding the modification of the organoleptic properties of food and the production of secondary compounds that can be harmful to health [2,5,6].

One of the most widely used materials in food and medical device packaging is polyethylene terephthalate (PET) due to its excellent mechanical properties and chemical stability under several conditions [7], availability, and low production cost. Therefore, PET is an ideal candidate for its use as antibacterial surfaces [8]. Approaches to providing antimicrobial activity can be classified into two types: the first can be achieved by incorporating antimicrobial agents into the polymeric matrix, and the second by modifying the surface and coating it with antimicrobial agents [9,10]. The latter approach is one of the most widely used since it significantly maintains the polymeric matrix’s original properties. This approach can be carried out through different methodologies, such as using covalently bonded coatings, which make them more stable in different physical–chemical environments. Antimicrobial coatings can be polymers from a natural or synthetic origin, providing new properties to the original material such as stimulus-response [11], bacteriostatic effects [12], biocompatibility [13], or allowing the loading and stabilization of nanomaterials [14]. In this context, chitosan, which is a polymer of natural origin, has shown antimicrobial activity against different pathogenic microorganisms with a high mortality rate and low toxicity to humans in various studies, making this polymer suitable for different applications in food [15], drug-delivery [16,17], biomedical devices [18,19], and other chemistry fields [20]. Chitosan is also used to stabilize and load nanoparticles synthesized using different methods [21,22] and to add new functional properties to coated nanoparticles by using chitosan as a carrier, such as in magnetic drug delivery systems [23,24]. Moreover, some nanoparticles are capable of providing antimicrobial activity through the release of metallic ions that affect the integrity of microorganisms, causing their death, as is the case with silver nanoparticles [25].

Chitosan has been widely modified to meet various biological and medical needs due to its active functional groups. Chemical modification is the commonly used method because its amino groups can participate in chemical reactions such as alkylation, quaternization, and condensation reactions with aldehydes and ketones. The hydroxyl group also gives rise to hydrogen bonding and some reactions, such as o-acetylation, cross-linking, and grafting [26,27]. Currently, the condensation reaction between the amino group and the carboxyl group is mainly used to modify the chemical properties of chitosan, improving its effect on metal nanoparticles, and reducing its possible toxic effects [28,29,30].

Chitosan is used as a coating on different materials, such as polyethylene terephthalate (PET), which is a slightly polar material and lacks active functional groups, making the adhesion, coating, or covalent grafting of chitosan difficult. In this research work, the surface of PET films was modified to perform the covalent grafting of chitosan, providing a suitable surface for loading silver nanoparticles. Additionally, the grafted chitosan chains possibly provide a bacteriostatic effect and a sustainable release of silver ions capable of inhibiting the proliferation of microorganisms and increasing the cytocompatibility of the final material.

## 2. Materials and Methods

### 2.1. Materials

Silver nitrate (99.9%), chitosan (75–85% deacetylated; 50–190 kDa), itaconic acid (99%), acetic acid, sodium carbonate, hydrochloric acid, ethylenediamine, and polyethylene terephthalate (PET) films (200 μm thickness; average molecular weight 18 kDa; 34.3% crystallinity) were purchased from Aldrich Chemical Co. (St. Louis, MO, USA). Ethanol and distilled water were acquired from Baker (Mexico City, Mexico).

### 2.2. Methods

#### 2.2.1. Aminolysis Reaction (PETN)

Three previously weighed PET films were placed in 10 mL of ethylenediamine at a temperature of 40 °C and reacted for different reaction times (0.5–4 h). Subsequently, the films were sonicated for 1 h and thoroughly washed with distilled water, removing the abraded surface of the film and residues from the reaction medium. Experiments were carried out in triplicate. The loss percentage was calculated according to the following equation (Equation (1)):

Weight loss (%) = 100[(W_PET_ − W_PETN_)/W_PET_] (1)

where W_PET_ is the weight of the unmodified PET film and W_PETN_ is the weight of the film once it has undergone the aminolysis reaction.

The quantification of the amino groups present on the surface of the film was carried out by acid-base titration. Three aminolysis reaction (PETN) films were placed in 10 mL of a hydrochloric acid solution [0.02 M], keeping them constantly stirred for 24 h. Subsequently, the excess of hydrochloric acid was titrated with a sodium carbonate solution [0.001 M]. The number of amino groups was calculated according to the following equation (Equation (2)):

Ethylenediamine modification (%) = 100[6.1(V_HCl_C_HCl_ − 2V_T_C_NaC_)/W_PETN_] (2)

where V_HCl_ and C_HCl_ are the volume and concentration of the hydrochloric acid solution, V_T_ is the volume of the titration and C_NaC_ is the concentration of the sodium carbonate solution and W_PETN_ is weight of the PETN film.

#### 2.2.2. Michael Addition Reaction (PETI)

Three previously weighed PETN films were made to react at reflux for 48 h in 10 mL of ethanol and an excess of itaconic acid (200 mg). Subsequently, the obtained films were exhaustively washed with distilled water. The incorporated itaconic acid was calculated according to the next equation (Equation (3))

Itaconic acid modification (%) = 100[(W_PETN_ − W_PETI_)/W_PETN_] (3)

where W_PETN_ is the weight of the PETN film and W_PETI_ is the weight of the film after the Michael addition reaction.

#### 2.2.3. Chitosan Grafting (PETC)

Three previously weighed Michael addition reaction (PETI) films were placed in 10 mL of an aqueous solution of chitosan 1% (*w*/*v*) and 0.5% (*v*/*v*) acetic acid for 48 h at 25 °C. After this time, the films were removed from the solution and placed under vacuum at a temperature of 90 °C for 6 h. Finally, the obtained films (PETC) were exhaustively washed with distilled water and dried.

The percentage of chitosan graft on the surface of the film was calculated according to the following equation (Equation (4)):

Chitosan graft (%) = 100[(W_PETI_ − W_PETC_)/W_PETI_] (4)

where W_PETI_ and W_PETC_ are the weight of the PETI and PETC films.

#### 2.2.4. Load of Silver Nanoparticles on PETC Film (PETCAg)

Three previously weighed PETC films were placed in 10 mL of an aqueous solution of silver nitrate with different concentrations (500, 1000, 3000, and 5000 ppm) for 72 h at 25 °C in the presence of sunlight. After the reaction time, the films were removed from the solution and thoroughly washed with distilled water and dried. The number of nanoparticles loaded on the PETCAg film was determined by film calcination using the weight difference of the residues yield at 800 °C (Equation (5)).

Silver load (%) = C_PETAg_ − C_PETC_ (5)

where C_PETCAg_ and C_PETC_ are the residue percentage of the PETCAg and PETC films provided by the TGA instrument.

#### 2.2.5. Cell Viability Study

Cell viability tests were performed using a BALB/3T3 fibroblast line (mouse). These experiments were performed to assess the toxicity of the films in an in vitro model for potential biomedical applications. For the experiments, 25 mg of each type of film were cut and placed in 96-well plates containing 3000 cells (fibroblasts), with Dulbecco’s modified Eagle’s medium (DMEM), penicillin–streptomycin, gentamicin, and Fetal Bovine Serum (FBS); the films were in contact with the cell medium for 24 h in an incubator at 37 °C (5% CO_2_). Subsequently, the films were removed from the cell medium, and MTT kit reagent (Roche, Switzerland) was added and incubated again for 4 h; later, they were solubilized and incubated for 12 h. The 96-well plates were plated in a Multiskan FC spectrophotometer, Thermo Fisher Scientific (Waltham, MA, USA); the absorbances from each film were read at a wavelength of 620 nm. Finally, cell activity (viability) was determined, making a comparison with control cells, using the following equation (Equation (6)), and the results obtained were statically analyzed by analysis of variance (ANOVA) using Fisher’s comparison (Figures S1–S7, Tables S1–S4):

Cytocompatibility (%) = 100(Abs_Sample_/Abs_Control_) (6)

### 2.3. Instrumental

Kruss DSA 100 drop shape analyzer (Matthews, NC, USA) was used to measure the contact angle on the surface of films at time 1 min.

Fourier Transform Infrared Attenuated Total Reflection (FTIR ATR) of dry samples was analyzed using a Perkin–Elmer Spectrum 100 spectrometer (Norwalk, CT, USA) of 16 scans.

Scanning electron microscope (SEM) images were acquired by the Zeiss Evo LS15 instrument (Jena, Germany), and small pieces of 0.5 cm in length were cut and coated with gold and analyzed under a high vacuum.

Thermogravimetric analysis (TGA) data of the weight loss and decomposition of films were heated at a rate of 10 °C min^−1^ and run from 20 to 800 °C under nitrogen flow at 100 cm^3^/min in a TGA instrument Q50 TA Instruments (New Castle, DE, USA).

Differential scanning calorimetry (DSC) runs were recorded under a nitrogen flow at 100 cm^3^/min using a DSC 2010 calorimeter (TA Instruments, New Castle, DE, USA) from 25 to 250 °C at a heating rate of 10 °C min^−1^.

Mechanical properties of the films were studied by applying a uniaxial tension test, as described in ASTM D1708. All tests were carried out on an INSTRON 1125 (Instron Inc., Norwood, MA, USA) universal tensile testing machine at a crosshead speed of 10 mm/min, and all experiments were carried out in triplicate.

## 3. Results and Discussions

### 3.1. Chitosan Grafting on PET Film

**Figure 1:**
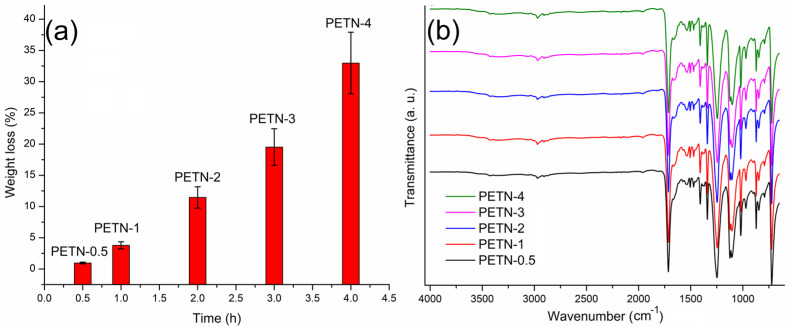
(**a**) Weight loss of PET films in the aminolysis reaction at different times, (**b**) IR-ATR spectra of PET films after aminolysis reaction at different times.

PET films were functionalized by aminolysis using ethylenediamine as a reagent and solvent. This reaction was studied at different times at a temperature of 40 °C. The results showed a weight loss of the PET film in the functionalization process with amino groups (Figure 1a). The weight loss increased with the reaction time. In the IR-ATR spectra (Figure 1b), the intensity bands of amines barely changed, where PETN-0.5 corresponds to the sample with the shorter time of reaction (30 min), while PETN-4 corresponds to the longer time of reaction (4 h). This result was confirmed in the acid-base titration (Table 1), since the increment in the number of amino groups on the film surface was small. When reaction times longer than 4 h were used, film disintegration occurred when the films were washed.

**Figure 2:**
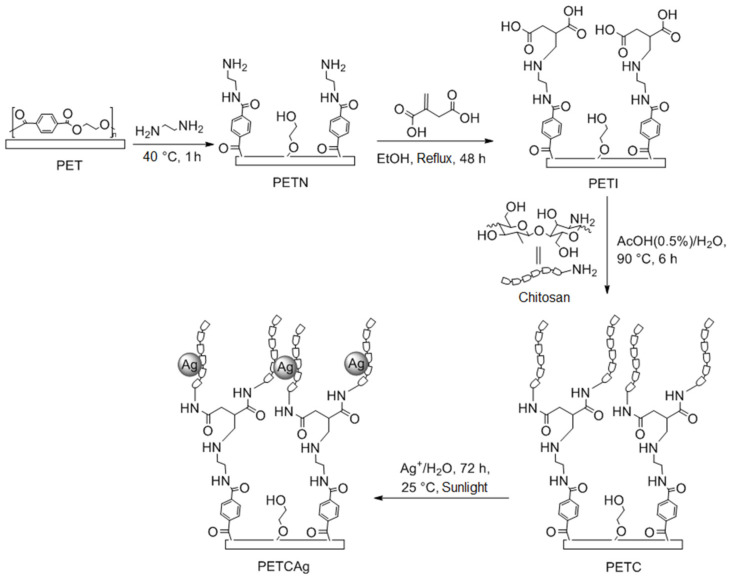
Scheme of chitosan graft on the functionalized PET film.

According to the results obtained at different reaction times and infrared studies, the modified films with a reaction time of 1 h were selected to continue with the next chemical activation step of the PET film, which presented an average amount of primary amino groups on the surface of 0.0078 mmol quantified by acid-base titration, with a percentage of the modification of the film with ethylenediamine by a weight of 1.25 ± 0.5%. The next activation step was carried out through the Michael addition reaction, between the amino groups present on the surface film and itaconic acid; this reaction was carried out in ethanol at reflux for 48 h. The resulting films were exhaustively washed with methanol and water, providing a weight-average surface modification of the 0.8 ± 0.2%. Subsequently, the obtained films were placed in 10 mL water at 5% acetic acid with 1% chitosan for 48 h to 25 °C. Finally, the films were removed from the chitosan solution and incubated at 90 °C for 6 h under vacuum, showing grafting of 1.38 ± 0.2% according to the weight of the chitosan. Once the films were obtained, they were placed in a solution of silver nitrate (3000 ppm) in the presence of sunlight to promote the reduction in silver ions and nucleation of silver to nanoparticles (Figure 2), resulting in an average load of 6.3 ± 1.3% by weight of silver nanoparticles concerning the matrix.

### 3.2. Contact Angle Study

**Figure 3:**
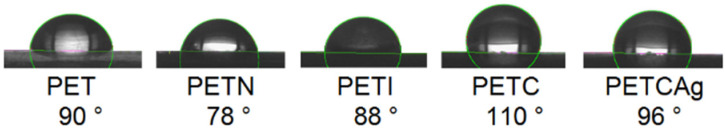
Contact angle of superficially modified films.

Contact angle studies showed changes in the hydrophilicity of the films. The PET films had a contact angle of 90° which decreased to 78° when modified with ethylenediamine; moreover, upon reaction with itaconic acid, the angle slightly increased to 88°. These results are in accordance with the new hydrophilic groups present on the film surface. When grafting the chitosan chains, the contact angle increased to 110°. Finally, the loading of the silver nanoparticles caused a slight increase in the hydrophilicity of the film (Figure 3).

### 3.3. FTIR-ATR Analysis

**Figure 4:**
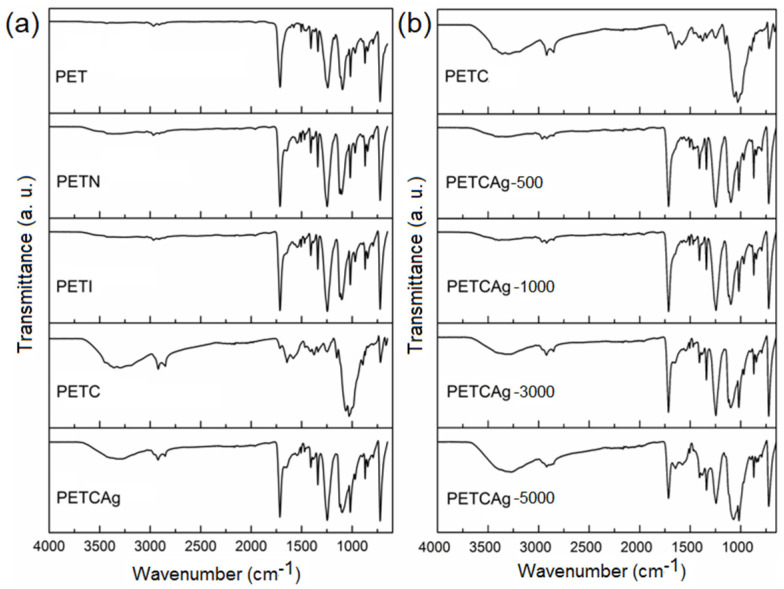
(**a**) FTIR-ATR spectra of PET films in the chitosan chain grafting process; (**b**) FTIR-ATR spectra of the PETC films loaded with silver nanoparticles in different silver nitrate concentrations.

TIR-ATR spectra (Figure 4a) were recorded to obtain information about the chemical groups incorporated into the PET films. The bands that evidenced the incorporation of the amino groups on the PET film surface were found at 3356 cm^−1^ (Figure 4a, PETN), which were assigned to the stretching vibration of the –NH_2_ and –OH groups, as well as the corresponding bands of the ester carbonyl groups at 1713 cm^−1^ and the amide carbonyl at 1676 cm^−1^. The spectra of the PETI film (Figure 4b, PETI) did not show the presence of double bonds, indicating the successful Michael addiction reaction and showing an increase in the band at 1724 cm^−1^, as well as a greater amplitude of the band located at 3356 cm^−1^ corresponding to the carboxylic groups from the addition of itaconic acid and the secondary amino groups formed after the reaction.

Spectra of the PETC film indicated an adequate grafting of the chitosan polymer chains on the functionalized PET film, showing a decrease in the bands corresponding to the carbonyl groups coming from the matrix due to the coverage of grafted chitosan and showing an increase in the bands corresponding to the –OH and –NH_2_ groups from the chitosan chains grafted at 3368 and 3299 cm^−1^ and amide carbonyl at 1645 cm^−1^, as well as the vibrations of the C–H bonds at 2850–2920 cm^−1^ and C–O–C at 1098–1121 cm^−1^. Finally, the load of silver nanoparticles caused a broad band at 3300 cm^−1^, probably due to the interactions of the –OH and –NH_2_ groups with the silver nanoparticles.

This study indicated that silver nanoparticles were obtained through a reduction in silver nitrate using chitosan. The FTIR of PETCAg shows the presence of new bands at 1715 and 1250 cm^−1^ due to the carboxylic and carbonyl groups confirming the oxidation of the hydroxyl groups of chitosan by the silver reduction. Likewise, the infrared spectrum shows that the carboxylic and carbonyl groups’ bands increase as the silver nitrate concentration increases (Figure 4b) [31,32].

### 3.4. Thermal Analysis

**Figure 5:**
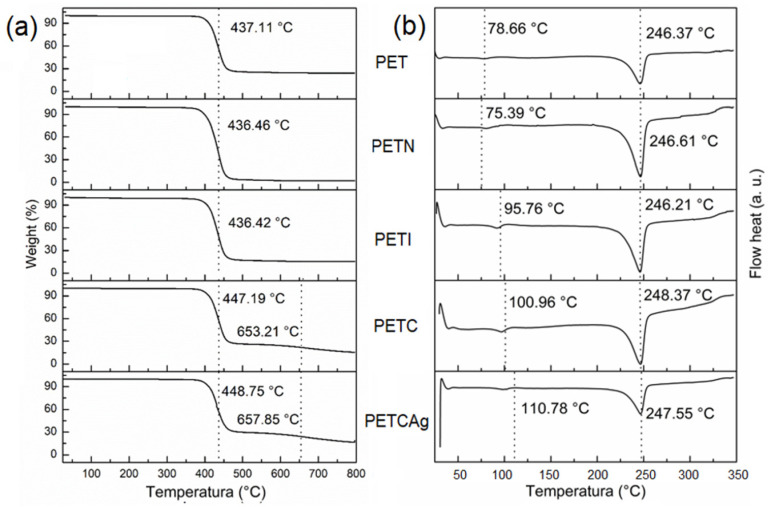
(**a**) TGA and (**b**) DSC studies under nitrogen flow on PET films obtained in the chitosan chain grafting process.

The thermogravimetric analysis (Figure 5a and Table 2) shows that the films have a decomposition temperature range of 436 to 448 °C. However, after 460 °C, PETC and PETCAg films show a second decomposition range, which is attributed to the decomposition of the chitosan grafted onto the film. The amount of the residue yield obtained is shown in Table 2. DSC studies show that the melting temperature of the PET film (246 °C) was not affected by the grafting process. The PET film’s glass transition (Tg) shows an increase in the grafting process. When itaconic acid was incorporated, the Tg of PETI increased from 78.6 to 95.6 °C; the addition of chitosan raised the Tg to 100.9 °C. Loading of silver nanoparticles elevated the Tg of the PETCAg to 110.7 °C, probably due to the strong interactions of the polymer chains with the silver nanoparticles (Figure 5b) [33].

**Figure 6:**
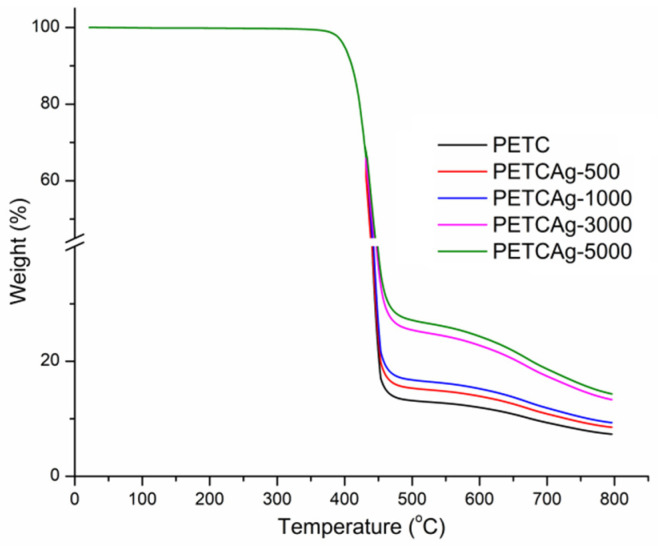
Silver quantification on PETCAg films by TGA under nitrogen flow.

Silver quantification in PETC films was carried out by residual weight at 800 °C. PETC films not exposed to a silver nitrate solution presented carbonization with an average residue of 7.33%. Therefore, the residual weight difference of the total mass of the films incubated in the silver solutions (500, 1000, 3000, and 5000 ppm) can be attributed to the silver nanoparticles loaded on films, resulting in a residual weight difference of 1.2 ± 0.4, 2.1 ± 0.6, 6.3 ± 1.3, and 7.1 ± 1.4%, respectively (Figure 6). As a result, a clear correlation was observed between the amount of silver nanoparticles and the concentration of silver nitrate used in the reaction medium [34].

### 3.5. Study of Mechanical Properties

**Figure 7:**
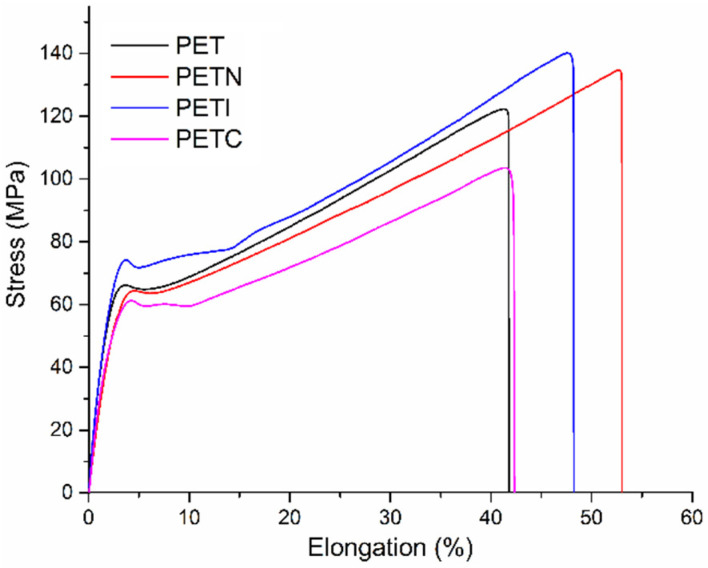
Tensile stress–strain graph of PET films in the process of grafting chitosan polymer chains.

The mechanical properties of the films were not significantly affected when grafting the chitosan polymer chains (Table 3), indicating that these were only grafted on the surface of the material, findings which were confirmed by the SEM studies, and the differences are possibly attributed to the wear of the material in the surface modification process, increasing the deformation for the PETN and PETI films, decreasing again, and becoming very similar to the original matrix for the films of PETC, with this finding being attributed to the grafting of the chitosan polymeric chains (Figure 7).

### 3.6. SEM Study

**Figure 8:**
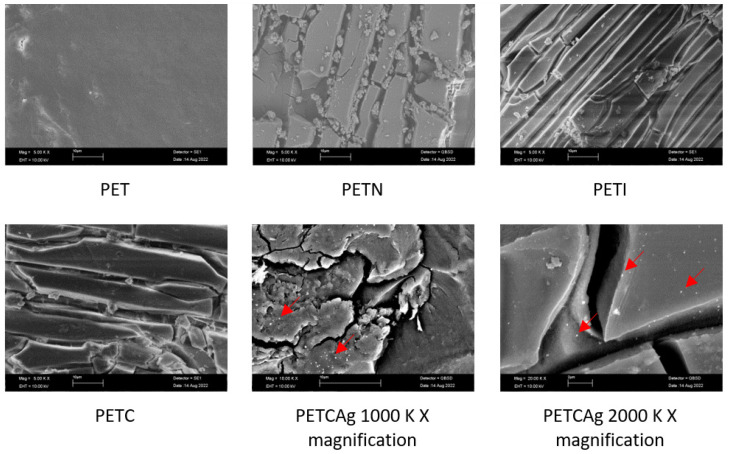
SEM images of PET films at different stages of the grafting process of chitosan polymer chains and with silver nanoparticles (marked with red arrows).

SEM studies of the modified films showed relevant changes with respect to the PET films (Figure 8). The magnification showed that the film modification process caused the formation of elongated structures in the form of layers across the surface. The formation of the layers and their thickness increased with the grafting process of the chitosan chains. Finally, the SEM images also showed the presence of silver nanoparticles on the formed chitosan layer with a mean size of 130 ± 37 nm.

### 3.7. Cell Viability Study

**Figure 9:**
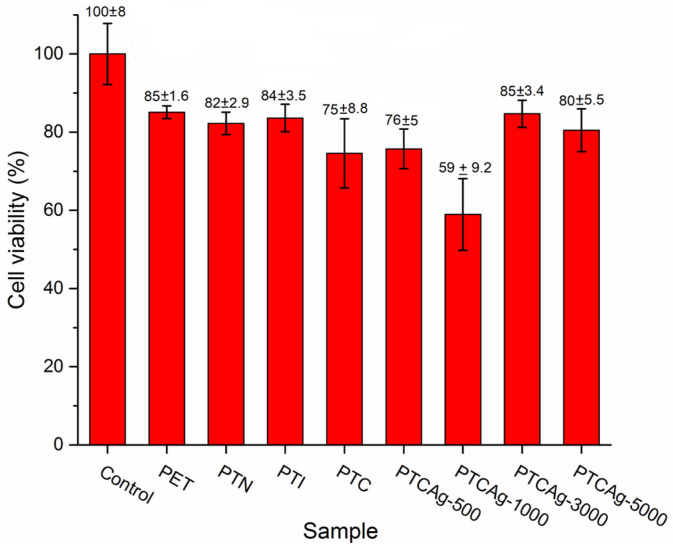
Cell viability along the grafting process (PET, PETN, PETI, and PETC) and silver-loaded PETC with different silver nanoparticles percentages (PETAg-500 (1.2 ± 0.4%), PETAg-1000 (2.1 ± 0.6%), PETAg-3000 (6.3 ± 1.3%), and PETAg-5000 (7.1 ± 1.4%)).

Cell viability was evaluated against 3T3 fibroblasts (Figure 9). The cell viability of the films decreased by 15–25% compared to the control, showing no differences among these samples, as indicated by statistical analysis (Figures S1–S7, Tables S1–S4). The exception was the PETCAg-1000 film, for which viability was 59 ± 9.2%. This finding indicates that the films, except for the PETC-1000 film, have good compatibility regardless of the modification and percentage of the silver loaded.

## 4. Conclusions

The grafting of chitosan polymer chains was successfully carried out by employing adequate surface functionalization of the PET films. The grafted chitosan allowed the nucleation and loading of silver nanoparticles with different percentages (1.2–7.1% with respect to the matrix) without using other reducing agents. Silver nanoparticles with an average size of 130 ± 37 nm were obtained. The films with a silver-coated surface obtained in this work are a good candidate for use as part of antimicrobial biomedical devices and disposable medical devices

## Figures and Tables

**Table 1:** Modification percentage of the PET film by the aminolysis reaction at different times, quantified by acid-base titration.

Time (h)	0.5	1	2	3	4
**Modification PET with ethylenediamine (% w.)**	1.18 ± 0.75	1.25 ± 0.5	1.31 ± 0.25	1.23 ± 0.37	1.39 ± 0.12

**Table 2:** Decomposition temperatures of PET films obtained in the chitosan chain grafting process.

Sample	Glass Transition Temperature (°C)	Decomposition Temperature (°C)	Residue Yield (800 °C, w.%)
PET	78.6	437.11	24.33
PETN	75.3	436.46	2.03
PETI	95.7	436.42	15.41
PETC	100.9	447.1, 653.21	7.33
PETCAg	110.7	448.75, 657.85	13.13

**Table 3:** Mechanical properties of PET films in the grafting process.

Sample	Elastic Modulus (MPa)	Stress Rupture (MPa)
**PET**	1144.762 ± 216.627	122.163 ± 20.3536
**PETN**	990.817 ± 104.047	136.780 ± 7.45101
**PETI**	1247.815 ± 188.311	140.172 ± 13.7654
**PETC**	1188.304 ± 122.115	103.506 ± 17.6539

## Data Availability

The data presented in this study are available on request from the corresponding author.

## Supplementary Materials

The following supporting information can be downloaded at: https://www.mdpi.com/article/10.3390/polym15010125/s1, Table S1: Analysis of Variance; Table S2: Model Summary; Table S3: Means; Table S4: Grouping Information Using the Fisher LSD Method and 95% Confidence; Figure S1: Fisher individual 95%; Figure S2: Interval plot of control PET; Figure S3: Individual value plot of control PET; Figure S4: Boxplot of control PET; Figure S5: Normal Probability Plot; Figure S6: Versus fits; Figure S7: Histogram of residual values.
