# Supplementary material for: Silver Nanoparticles Loaded on Polyethylene Terephthalate Films Grafted with Chitosan
Source: Polymers (Basel). 2022 Dec 28;15(1):125. doi: 10.3390/polym15010125 (PMC9824822; doi:10.3390/polym15010125)
Supplement: Supplementary file 1 [file polymers-15-00125-s001.zip › polymers-2085612-supplementary.pdf]

Supplementary Materials

Silver Nanoparticles Loaded on Polyethylene Terephthalate Films Grafted with Chitosan

Guadalupe Gabriel Flores-Rojas [1], Felipe López-Saucedo [2], Ricardo Vera-Graziano [3], Héctor Magaña [4], Eduardo Mendizábal [1,*] and Emilio Bucio [2,*]

[1] Departamento de Química e Ingeniería Química, Centro Universitario de Ciencias Exactas e Ingenierías, Universidad de Guadalajara, Blvd. M. García Barragán # 1451, Guadalajara 44430, Jalisco, Mexico
[2] Departamento de Química de Radiaciones y Radioquímica, Instituto de Ciencias Nucleares, Universidad Nacional Autónoma de México, Circuito Exterior, Ciudad Universitaria, Mexico City 04510, Mexico
[3] Instituto de Investigaciones en Materiales, Universidad Nacional Autónoma de México, Circuito Exterior, Ciudad Universitaria, Mexico City 04510, Mexico
[4] Facultad de Ciencias Químicas e Ingeniería, Universidad Autónoma de Baja California, Calzada Universidad # 14418, Parque Industrial Internacional Tijuana, Tijuana 22390, Mexico
* Correspondence: lalomendizabal@hotmail.com (E.M.); ebucio@nucleares.unam.mx (E.B.)

WORKSHEET 1

One-way ANOVA: Control, PET, PTN, PTC, PETC, PETCAg-500, PETCAg-1000, PET-CAg-3000, PETCAg-5000

Method

Null hypothesis	All means are equal
Alternative hypothesis	Not all means are equal
Significance level	α = 0.05

Equal variances were assumed for the analysis.

Factor Information

Factor	Levels	Values
Factor	9	Control, PET, PTC, PTI, PTN, PETCAg-500, PETCAg-1000, PETCAg-3000, PETCAg-5000

Table S1. Analysis of Variance.

Source	DF	Adj SS	Adj MS	F-Value	P-Value
Factor	8	2848.8	356.10	10.14	0.000
Error	18	632.3	35.13		
Total	26	3481.1			

Table S2. Model Summary.

S	R-sq	R-sq(adj)	R-sq(pred)
5.92681	81.84%	73.76%	59.13%

Table S3. Means.

Factor	N	Mean	StDev	95% CI
Control	3	100.00	8.00	(92.81, 107.19)
PET	3	85.100	1.600	(77.911, 92.289)
PTC	3	74.93	8.80	(67.74, 82.12)
PTI	3	83.60	3.50	(76.41, 90.79)
PTN	3	82.20	2.90	(75.01, 89.39)
PETCAg-500	3	75.80	5.00	(68.61, 82.99)
PETCAg-1000	3	59.00	9.20	(51.81, 66.19)
PETCAg-3000	3	85.00	3.40	(77.81, 92.19)
PETCAg-5000	3	80.00	5.50	(72.81, 87.19)

Pooled StDev = 5.92681

Fisher Pairwise Comparisons

Table S4. Grouping Information Using the Fisher LSD Method and 95% Confidence.

Factor	N	Mean	Grouping		
**Control**	**3**	100.00	A		
PET	3	85.100		B	
PETCAg-3000	3	85.00		B	
PTI	3	83.60		B	
PTN	3	82.20		B	
PETCAg-5000	3	80.00		B	
PETCAg-500	3	75.80		B	
PTC	3	74.93		B	
PETCAg-1000	3	59.00			C

Means that do not share a letter are significantly different.

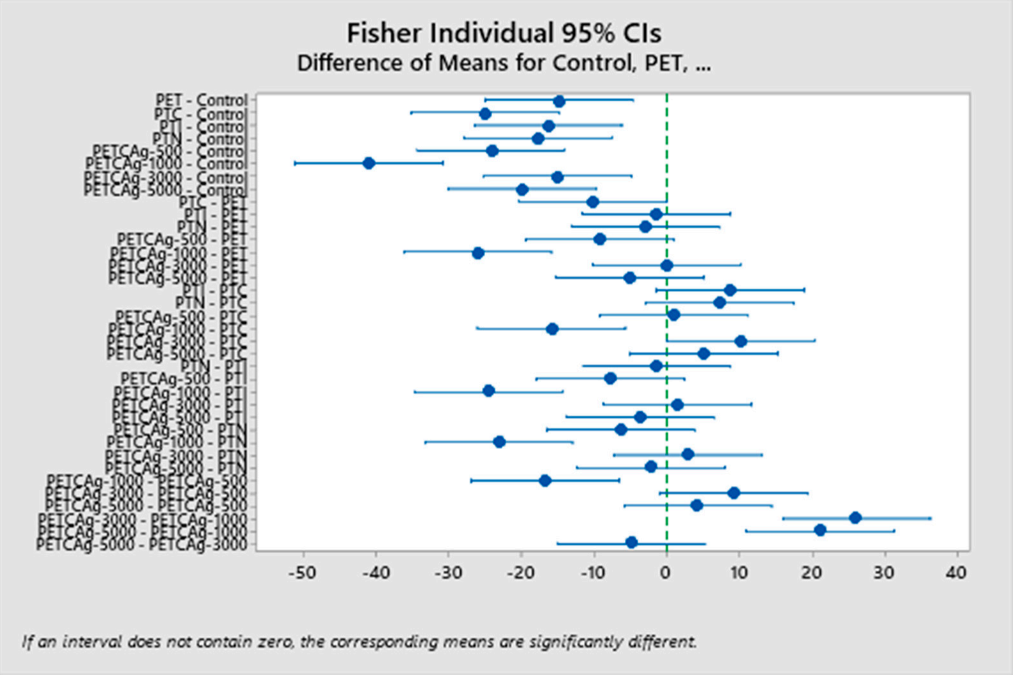

Figure S1. Fisher individual 95 %.

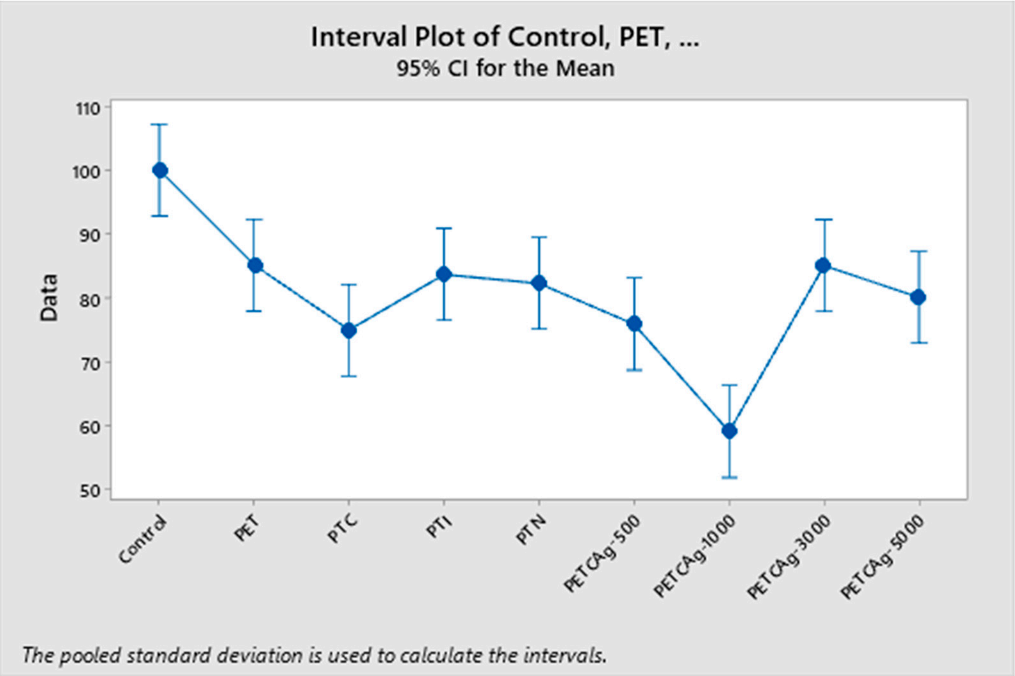

Figure S2. Interval plot of control PET.

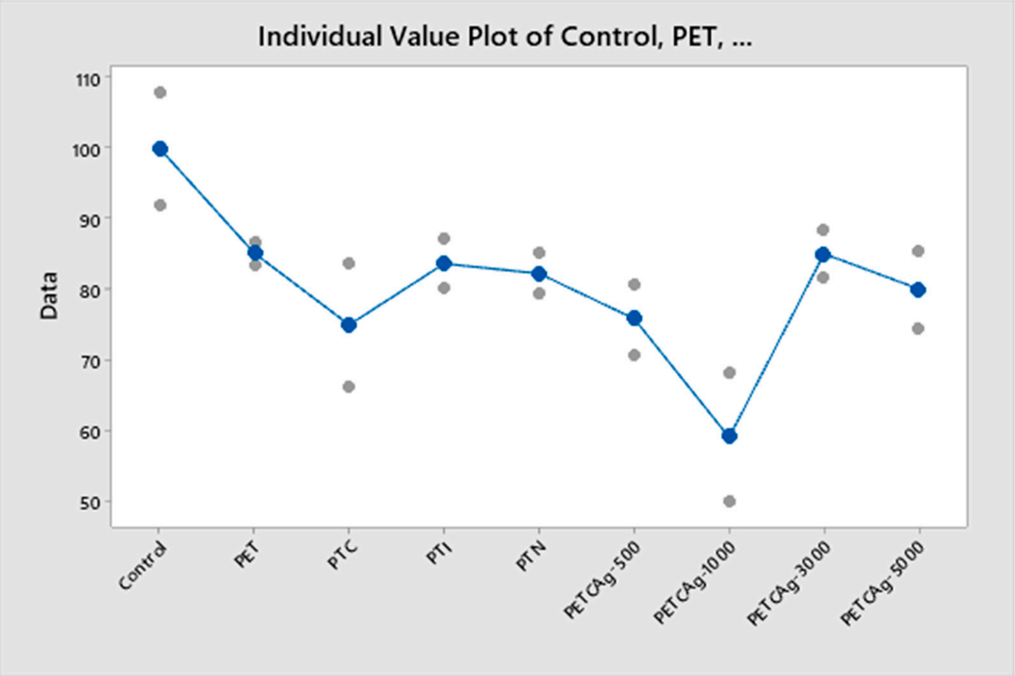

Figure S3. Individual value plot of control PET.

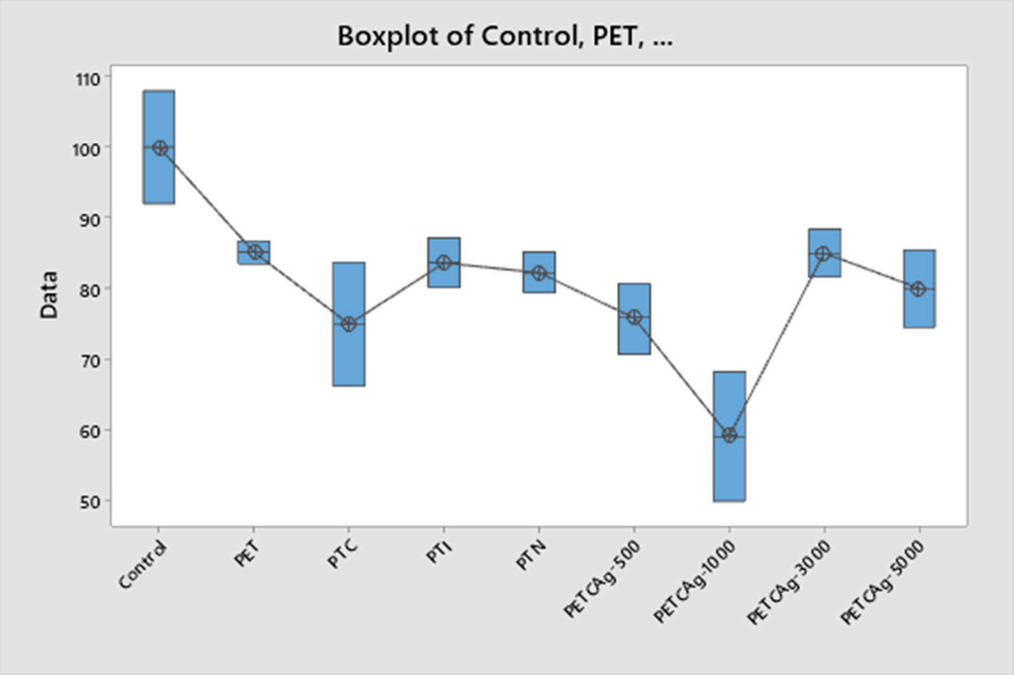

Figure S4. Boxplot of control PET.

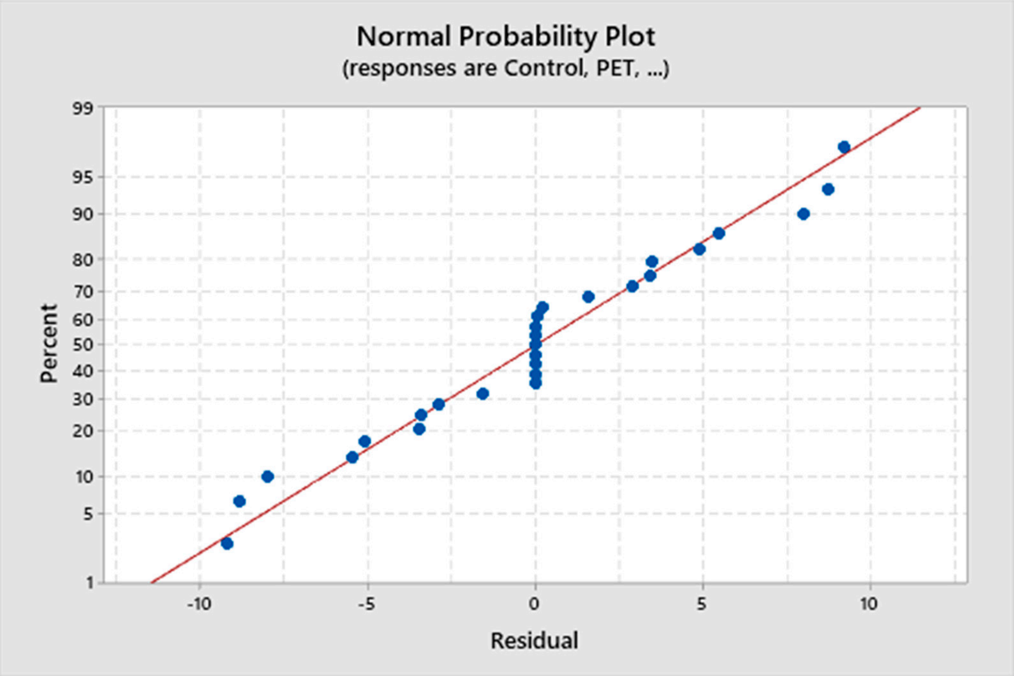

Figure S5. Normal Probability Plot.

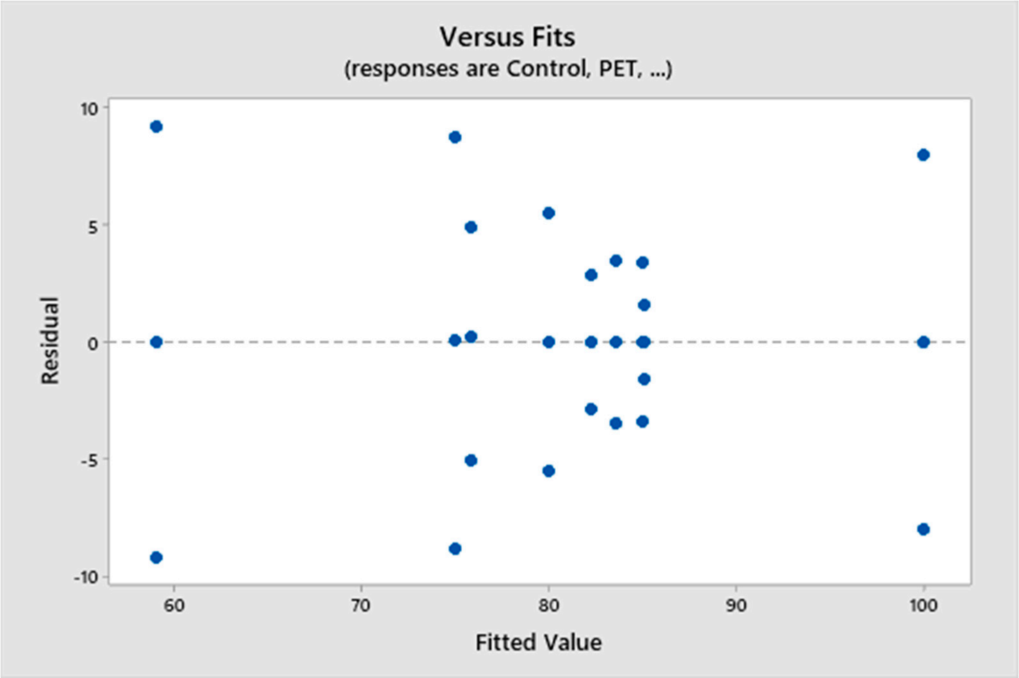

Figure S6. Versus fits.

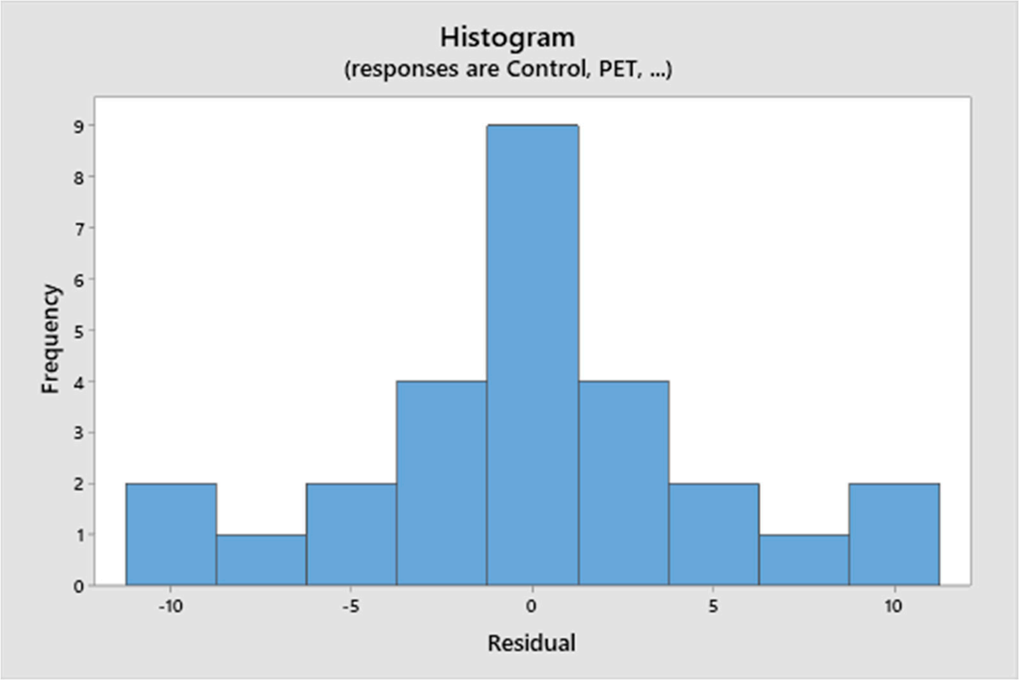

Figure S7. Histogram of residual values.
